# Proteomic analysis of HEK293 cells expressing non small cell lung carcinoma associated epidermal growth factor receptor variants reveals induction of heat shock response

**DOI:** 10.1186/s40164-015-0010-5

**Published:** 2015-06-12

**Authors:** Arpana Kamath, Ann M Joseph, Kumud Gupta, Digamber Behera, Anand Jaiswal, Ravindra Dewan, Maitreyi S Rajala

**Affiliations:** School of Biotechnology, Jawaharlal Nehru University, New Delhi, 110 067 India; National Institute of Tuberculosis and Respiratory Diseases, New Delhi, 110 030 India

**Keywords:** EGFR, NSCLC, TKI, Heat shock proteins, Tumor progression, Drug sensitivity

## Abstract

In view of the fact that certain non small cell lung carcinoma associated epidermal growth factor receptor mutations keep the receptor constitutively active, the downstream effectors of altered activity of mutant receptors are largely unknown. By 2D gel electrophoresis and MALDI-TOF/MS analysis, we showed that increased activity of EGFR mutants, L858R, L861Q and A871G induce heat shock proteins such as Hsp70, Hsp60, Hsp90B1, Hsp5a, Hsp71 and few transcriptional factors. Of which, Hsp70 was observed to be regulated more selectively to L861Q mutant. Our results suggest the possible role of heat shock proteins in lung tumor progression considering EGFR mutations.

**Electronic supplementary material:**

The online version of this article (doi:10.1186/s40164-015-0010-5) contains supplementary material, which is available to authorized users.

## To the editor

Large number of studies reported epidermal growth factor receptor mutations (EGFR) in non small cell lung carcinoma (NSCLC) patients worldwide, most commonly in Asian countries including India in the last decade [1, 2]. *In vitro* studies have demonstrated the contribution of EGFR mutations to uncontrolled tumor proliferation and evasion of programmed cell death in various cancers [3, 4] including lung tumorigenesis in transgenic mice models [5]. It is known that a set of NSCLC associated EGFR mutations especially in tyrosine kinase (TK) domain have accounted for to have prognostic significance as they sensitize the receptor to TKI [6, 7]. Therefore, the question remains unanswered why NSCLC tumors with certain mutations respond to targeted drugs while others make the tumor resistant to the same drug [8]. This implies the complexity of drug sensitivity in patients harboring EGFR mutations and the complexity may be due to altered activity of mutant receptors affecting various downstream molecules for tumor survival which are largely unknown. Till date, detection of EGFR mutations remains an important prognostic test, as FDA approved drugs including the drugs which are currently under development for NSCLC treatment target EGFR. Unfortunately, in spite of considering mutations for treatment prediction, prognosis of advanced stage tumors remains poor. At this juncture, identification of downstream effectors of altered mutant receptor activity with prognostic importance is essential. Uncovering of such molecules may also allow us to understand the lung tumor progression and complexity of drug sensitivity driven by receptor variants.

We initiated our study by screening FFPE lung tumor tissues derived from NSCLC patients from north Indian population for EGFR mutations by RT-PCR followed by sequencing after obtaining ethical approval and informed consent from patients. Detailed methodology was given in Additional file 1. Receptor activity and drug sensitivity was determined by measuring phosphorylation on tyr1068 residue of each mutant generated by site directed mutagenesis. Two amino acid substitutions, L861Q (10.5 %) and A871G (2.1 %) in exon21 and K879R (24.2 %) in exon22 of TK domain were detected (Additional file 2). Former two demonstrated increased receptor activity (Additional file 3), and sensitivity to TKI, Gefitinib more selectively (Additional files 4 and 5). While the latter one was found to be indistinguishable from wild type receptor with respect to its activity and drug sensitivity.

**Fig. 1:**
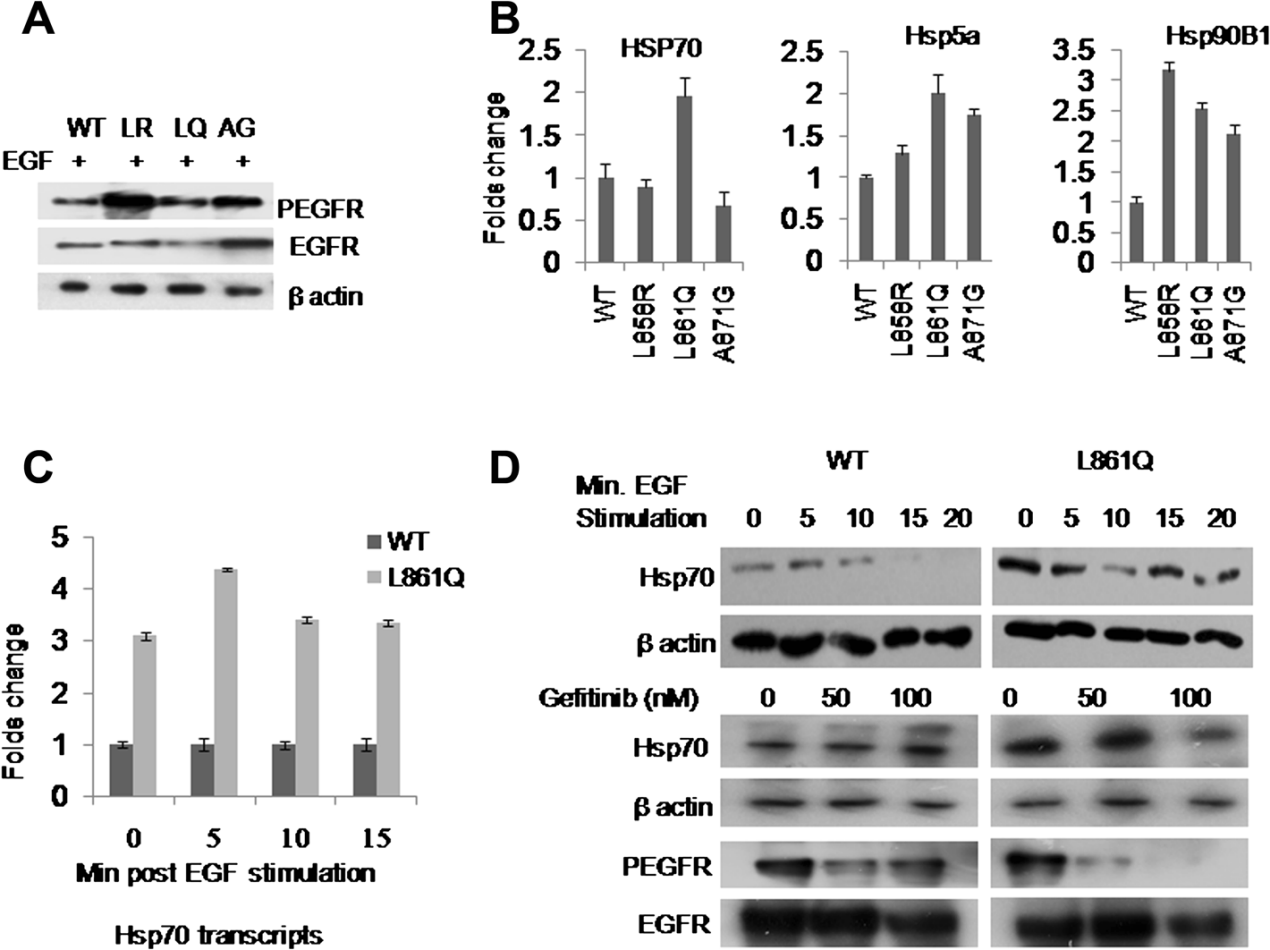
Induction of heat shock response to EGFR mutants and regulation of Hsp70 by L861Q mutant. HEK293 stable cell lines expressing each mutant and wild type receptor were seeded in 35 mm dishes in duplicates. At 80-90 % confluency, cells were serum starved overnight followed by EGF stimulation at 10 ng/ml. At 10 min post stimulation, cells were harvested. Total protein from one set of plates and total RNA from duplicate set of plates were recovered. Equal amount of protein recovered from each receptor expressing cells was subjected to western blot analysis using anti phospho EGFR antibody specific to autophosphorylation site, tyr1068 residue. Blot was striped and re-probed with total EGFR antibody. RNA samples were reverse transcribed and subjected to quantitative real time PCR using gene specific primers. For Hsp70 expression studies, L861Q mutant expressing cells were cultured, serum starved followed by EGF stimulation. At every 5 min interval, total RNA and protein lysates recovered were subjected to quantitative real time PCR using Hsp70 primers and western blot analysis with anti Hsp70 antibody respectively. For inhibitory studies; cells were serum starved, treated with different concentrations of Gefitinib followed by EGF stimulation. Western blot analysis was done using anti Hsp70, anti phospho and total EGFR antibodies. **a** Phosphorylation status of three mutants *vs.* wild type receptor. **b** Folds difference of mutants *vs.* wild type receptor regulated gene transcripts of few heat shock proteins. **c** Hsp70 expression at transcript level measured at every 5 min following EGF stimulation in L861Q mutant *vs.* wild type receptor expressing cells **d** Hsp70 expression at protein level for 20 min, Hsp70 and phospho EGFR levels in cells treated with TKI, Gefitinib

Further, we investigated proteomic profile of HEK293 cells in response to altered activity of mutants L861Q, A871G and the most widely reported mutation, L858R by 2D GE followed by MALDI-TOF/Mass spectrometry analysis. Representative 2D gel with resolved protein spots of wild type EGFR expressing cells and different densities of analyzed spots of wild type *vs*. mutant receptors were shown in Additional files 6 and 7. Protein profile of cells expressing each mutant upon EGF stimulation was compared with protein profile of wild type receptor. Heat shock proteins such as Hsp70, Hsp71, Hsp90B1, Hsp60, and Hsp5a were identified to be differentially regulated largely in response to EGFR mutants (Table 1). Other effectors were FGG, IFIT2, cytoskeletal proteins and transcriptional factors such as HOXD11, HOX B4 (Additional file 8). Most of the proteins identified herein including IFIT2 and FGG were reported to be associated with various cancers with the potential to metastasize the tumor [9, 10]. Few of the identified proteins were validated at transcript level by quantitative real time PCR (Fig. 1a, b, Additional files 9 and 10). Up regulation of Hsp70 more selectively to L861Q mutant activity was consistent in our experiments. Reduction of its expression with Gefitinib treatment (Fig. 1c and d) suggests the possible role of this mutant in Hsp70 regulation. Published literature strongly argues that certain molecular chaperones, more importantly Hsp70 play a significant role in tumor survival [11]. Hsp70 expression was already reported in primary NSCLC tumors [12] and serum samples collected from NSCLC patients [13] as well. However, we demonstrated first time that heat shock proteins are the major downstream effectors of NSCLC associated EGFR variants. Supporting our data, some proteins detected in our study were recently reported to be differentially expressed in a proteomic study carried out on interstitial fluids collected from NSCLC patients [14]. Considering the importance of molecular chaperones in tumor survival and with their change of expression in response to altered EGFR activity, we hypothesize that they may also regulate progression of NSCLC tumors harboring EGFR mutations. So, the molecular mechanism involved in tumor progression, drug complexity and the prognostic implications of these heat shock proteins in NSCLC patient management are worth exploring.

**Table 1:** Differentially expressed proteins in cells expressing mutants vs. wild type EGFR identified by MALDI-TOF/MS analysis

Spot No.	Protein name	Acc. No	Mol. Wt	PI	Mascot Score	L858R*vs.* WT	L861Q *vs.* WT	A871G *vs.* WT
6601	Heat shock cognate 71 kDa protein	P11142	71082	5.37	24	1.8	1.2	1.13
7501	Heat shock 70 kDa protein 1	P08107	70294	5.48	147	1.7	2.1	0.93
3702	Endoplasmin (GRP94) HSP90B1	P14625	92696	4.76	88	0.02	0.28	0.02
5503	60 kDa heat shock protein, mitochondrial	P10809	61187	5.7	75	1.4	1.2	1.03
4701	78 kDa GRP (HSP5A)	P11021	72402	5.07	158	1.1	1.31	0.34
7502	T-complex protein 1 subunit epsilon	P48643	60089	5.45	63	0.29	0.36	0.56
7403	Protein disulfide-isomerase	P30101	57146	5.98	84	0.73	0.96	0.63
4501	Tubulin alpha-1B chain	P68363	50804	4.94	128	0.58	0.69	0.33
3401	Tubulinbeta-2C chain	P68371	50255	4.79	239	0.63	1.2	0.72
4501	Vimentin	P08670	53676	5.06	24	0.58	0.69	0.33
6302	Keratin, type I cytoskeletal 18, 19	P05783	48029	5.34	319	0	1.5	0.86
7402	Keratin, type II cytoskeletal 8	P05787	53671	5.52	59	0.73	0.96	0.63
5203	Actin, cytoplasmic 2	P63261	42108	5.31	107	1.2	1.31	0.79

## Additional files

Additional file 1:
**Methodology.**
Additional file 2:
**EGFR mutation analysis on lung tumor tissues.**
Additional file 3:
**Ligand dependent phosphorylation of EGFR mutants.**
Additional file 4:
**Sensitivity of mutant receptors to Gefitinib.**
Additional file 5:
**Sensitivity of mutant receptors to Erlotinib.**
Additional file 6:
**2D gel with resolved proteins of EGFR expressing cells.**
Additional file 7:
**Different densities of protein spots in cells expressing mutants vs. wild type receptor.**
Additional file 8:
**List of proteins differentially regulated in response to mutant L861Q vs. wild type receptor identified by MS analysis.**
Additional file 9:
**List of real time PCR primers.**
Additional file 10:
**Quantitative real time PCR of regulated gene transcripts in cells expressing EGFR mutants.**
