# Supplementary material for: Proteomic analysis of HEK293 cells expressing non small cell lung carcinoma associated epidermal growth factor receptor variants reveals induction of heat shock response
Source: Exp Hematol Oncol. 2015 Jun 12;4:16. doi: 10.1186/s40164-015-0010-5 (PMC4490733; doi:10.1186/s40164-015-0010-5)
Supplement: Additional file 1: — Methodology. [file 40164_2015_10_MOESM1_ESM.pdf]

Methods

Detection of EGFR mutations in NSCLC patients

Patient samples

Tumor tissues derived from patients attending lung cancer clinic at National Institute of Tuberculosis and Respiratory diseases, New Delhi were included in this study. The project was approved by institutional ethical committee and the informed consent was taken from patients prior to sample collection. Patients were selected by physician based on radiological and clinical findings. Pathological evaluation of tissue sections was done and the representative tumor area was marked on H&E stained sections. Formalin fixed paraffin embedded tissue sections from clinically proven 95 NSCLC patients were analyzed. Non cancerous lung tissues collected from patients with other lung diseases were used as controls. To check the distribution of mutations between tumor and normal areas, on a single H &E stained section, more than one tumor area and histologically normal areas surrounding tumor area were selected. Cells were scrapped from each area and analyzed separately for mutations. However, separate analysis of paired normal and tumor cells was carried out on random samples. None of the patients received EGFR selective drugs, Gefitinib or Erlotinib prior to sample collection.

Primers

Two sets of primers were designed to amplify genome sequence of six exons encoding EGFR tyrosine kinase domain. First set of primers, both external and nested (External primers: FP- 5' CCTAAGATCCCGTCCATCG 3’, RP: 5' CTGCGGTGTTTTCACCAGTA 3', Nested Primers, FP: 5' CCTCTTGCTGCTGGTGGTGG 3', RP: 5' GCACCAAGCGACGGTCCTC 3') were used to amplify the region spanning exons18 to20. While second set of primers (External primers- FP:

5' GCATCTGCCTCACCTCCAC 3', RP: 5' AGGTAGCGCTGGGGGTCT 3' and Nested primers FP: 5' GCTGCCTCCTGGACTATGTCC 3', RP: 5' GAATTCGATGATCAACTCACGG 3') amplified the region spanning exons21 to 23 of kinase domain.

Reverse transcription-PCR amplification

Cells from marked tumor area on H&E stained sections were scrapped and total RNA was extracted by single step RNA extraction Guanidiniumisothiocyanate (GITC) method. A fraction of total RNA was reverse transcribed with 40U MMLV Reverse transcriptase, 5μM Oligo (dT)^{18} primer, 20U Ribonuclease inhibitor followed by amplification of cDNA using PCR mix containing 5X Kapa HiFi fidelity buffer with $MgCl_2$, 0.2mMdNTPs, 1U Kappa HiFi DNA polymerase (Kappa Biosystems Inc, MA) and 10pmols of primers specific for exons encoding EGFR kinase domain. Purified amplicons were sequenced in both forward and reverse direction for mutation analysis. Sequence variation was determined by NCBI blast software against EGFR gene sequence (accession no NM_005228.3).

Functional analysis of EGFR mutants

Generation of EGFR mutants and stable cell lines

EGFR mutants were generated by inserting single base changes at 2582ntT<A (L861Q), 2612nt C<G(A871G), and 2636A<G (K879R) into full length EGFR coding sequence in CMV promoter driven eukaryotic expression vector pcDNA3.1 using Quick Change Lightning Site-Directed Mutagenesis Kit (Stratagene, CA) following manufacturer’s instructions. Mutants were sequenced to confirm the alterations at desired positions. HEK 293 cell line with no endogenous EGFR expression was stably transfected with each of wild type and mutant receptors using

Lipofectamine 2000 (In vitrogen Life technologies, CA) according to manufacturer's instructions. Stable clones were selected with G418drug at a concentration of 600µg/ml. Expression of wild type and mutant receptors in stable cell lines was confirmed by western blot analysis using anti EGFR antibody (Santacruz Biotechnology Inc, USA). L858R mutant (2573T>G) was also generated as described above for its inclusion in proteomic study.

Phosphorylation of EGFR mutants

HEK293 cells expressing mutants and wild type receptors were serum starved overnight followed by EGF stimulation at 10ng/ml concentration for 10min. Total protein lysates were recovered from cells expressing mutants or wild receptors using 1X Lysis buffer containing 20mM Tris-HCl (pH 7.5), 150mM NaCl, 1mM Na2EDTA, 1mM EGTA, 1% Triton, 2.5mM sodium pyrophosphate, 1mM beta-glycerophosphate, 1mM Sodium Vanadate, 1 µg/ml leupeptin and 1mM PMSF (Cell Signaling Technology, USA). Protein concentration was determined and equal amount of protein from each sample was subjected to SDS PAGE followed by western blot analysis using phospho-specific EGFR (PY1068) antibody. Blots were stripped and re-probed with total EGFR antibody.

Sensitivity of EGFR mutations to known tyrosine kinase inhibitors

Purified compounds of tyrosine kinase inhibitors Gefitinib and Erlotinib (Biovision Inc; CA) were reconstituted in DMSO. MTT assay was performed to determine the toxic effects of drugs on HEK293. Wild type and mutant receptor expressing cells were serum starved overnight followed by drug treatment for 60 min with different concentrations ranging from 25nM to

200nM. At one hour post drug treatment, cells were stimulated with EGF at 10ng/ml; protein lysates were prepared and analyzed for receptor kinase phosphorylation as described above.

Proteomic analysis of HEK293 cells expressing EGFR mutants

Preparation of samples for IEF

Proteome analysis of HEK293 cells expressing EGFR mutants L861Q, A871G and L858R independent of each other was done and compared with protein profile of wild type receptor expressing cells. Six well plates seeded with HEK293 cells stably expressing each of wild type or mutant receptors (L858R, L861Q and A871G) were cultured for 24hrs in DMEM (Hi Media Laboratories) supplemented with 10% FBS. At ~90% confluence, cells were serum starved overnight; next day cells were washed once with serum free medium and stimulated with 10ng/ml EGF. At 10min post EGF stimulation, cells were washed thrice with ice cold 1X TBS (pH 7.6) and incubated on ice by adding 100µl of 2D extraction buffer (7M Urea, 2M Thiourea, 2% CHAPS, 20mM Tris- HCl (pH-7.6), 5mM DTT, 1mM PMSF, 1mM Sodium orthovanadate, 20units/ml DNases ,0.25mg/ml RNases, 0.2% Bio-Lyte® 3/10 and 1X protease inhibitor cocktail) for 30mins. Cells were scrapped and subjected to repeated freeze thaw cycles; cell lysates were collected into low bind protein tubes and centrifuged at high speed for 30min. Supernatants were collected and protein concentration was determined by Bradford assay.

Isoelectric focusing (IEF)

Protein lyasate of 169µg was mixed with rehydration buffer (8M urea, 2% CHAPS, 50mM DTT, 0.2% Bio-Lyte® 3/10 ampholyte, 0.001% Bromophenol Blue) to a final volume of 200µl. Lysates were loaded on to IPG strips of 7cm of linear broad pH range of 3-10 or narrow pH

range of 4-7. Strips were rehydrated for 16 hours at room temperature. Next day, rehydrated strips were placed in an IEF tray and subjected to IEF to separate protein spots under following conditions; 250Vrapid (1 hour);500V rapid (30 minutes);1000V rapid (30 minutes);4000V linear (30 minutes) ;4000V rapid ,up to 12,000 Volt-hr (for IPG strip pI 3-10); 15,000 Volt-Hr (for IPG strip pI 4-7).

SDS PAGE

Following IEF, IPG strips were equilibrated in equilibration buffer I (375mM Tris- HCl (pH 8.8), 6M urea, 2% SDS) containing 2% DTT for 15min. After reduction, equilibration buffer I was replaced with equilibration buffer II containing 2.5% iodoacetamide for 15min. IPG strips with separated proteins were subjected to second dimension SDS PAGE followed by staining with gel code safe blue (Thermo Scientific Co, USA). Imaging of gels was done on phosphor-Imager (Bio-Rad, USA) using quantity one software and protein spots on the gels were analyzed by PD Quest Software (Bio-Rad, USA). Protein spots with significant difference in fold intensity between mutants vs. wild type receptor gels as determined by PD Quest software were selected for identification by MALDI-TOF/MS analysis. Selected spots were excised using 2D gel spot cutter (Bio-Rad, USA) and gel plugs were subjected to tryptic digestion.

Trypsin Digestion of 2D Spots (In gel digestion)

Excised gel plugs were digested with trypsin prior to MALDI-TOF MS/MS analysis. In brief, gel plugs were cut into small pieces and briefly rinsed with 25mM ammonium bicarbonate (ABC) and dehydrated with 2:1 ratio of acetonitrile (ACN) and 50mM ABC for 5min followed by rehydration in 25mM ABC for 2min. The process of dehydration and rehydration was done

twice followed by reduction with 10mM DTT for 1 hour at 60°C and alkylation with 55mM iodoacetamide (IAA) for 20min at RT in dark. Then, gel pieces were subjected to three more rounds of dehydration and rehydration as described above. Gel pieces were digested over night with 20ng/µl trypsin (Sigma) in 25mM of ABC at 37°C. Digested peptides were extracted by re-suspending gel pieces in 50% ACN: 1% trifluoroacetic acid (TFA) and sonicated for 10min followed by centrifugation. The extraction step was done one more time and all the supernatants obtained after every extraction step were pooled and subjected to speed vacuum and re-suspended in 50%ACN: 0.1% TFA.

MALDI-TOF/MS analysis

Digested peptides were loaded onto 384 OptiTOF- MALDI plate (Applied Bio systems) by mixing 0.5µl of sample with 0.5µl of 1 % α-Cyano-4-hydroxycinnamic acid (CHA). Spots were air dried and loaded on to proteome analyzer SCIEX TOF/TOF 5800 (Applied Bio systems). The peptides identified by MALDI-TOF/MS were further subjected to MS/MS fragmentation. TOF/TOF ™ series explorer ™ Software was used for acquisition and processing of MS/MS spectra. The identified peptides were searched against MASCOT database using Protein Pilot software (AB SCIEX) connected to its server. Parameters were set, such as variable modifications (Deamidated, Oxidation), fixed modifications (Carbamidomethyl), peptide mass tolerance (±250ppm), fragment tolerance (±0.3Da) with maximum missed cleavage up to 1/2.The protein hits were obtained for all the spots that were selected from 2D gels for protein identification by MS analysis.

Differential proteomics and validation by quantitative PCR

Real time PCR and data analysis

As inferred by PD Quest Analysis Software, proteins that were differentially expressed in WT *vs.* mutant receptors expressing cells were validated at transcript level by Quantitative Real Time PCR. Primers were designed using corresponding nucleotide sequences by primer express (3.0) software (Applied Biosystem). Primers sequences are listed in Additional file 9.

As described above in the phosphorylation experiments, stable cells expressing EGFR mutants were cultured, serum starved, and stimulated with EGF. At 10min post EGF stimulation, total RNA was isolated and reverse transcribed using Verso cDNA synthesis kit (Thermo Scientific, USA) in a reaction mixture containing 1µg total RNA, random primers, RT enhancer, RT enzyme, dNTPs and RNase inhibitors. The reaction mixture was incubated at 42°C for 30min followed by 95° C for 2min. Two µl (100ng) of cDNA was used for real time PCR amplification using SYBR® Green chemistry (KAPA Biosystem, Saf labs). Real time PCR was carried out in 25 µl reaction mixture containing SYBR® DNA polymerase, SYBR® Green I Fluorescent dye, $MgCl_2$, dNTPs and stabilizers, 10µM of forward and reverse primers. GAPDH gene amplification was used as internal control for normalization. Each sample including non template controls was amplified in triplicates.

Relative Quantification by Comparative $\Delta\Delta C_T$ Method

Real time data was analyzed using comparative $\Delta\Delta C_T$ method. Fold changes of target transcript levels of corresponding proteins of mutants *vs.* wild type receptor expressing cells was calculated with obtained C_T values using the formula $\mathbf{2^{-\Delta CT\,(mutant)\,-\,\Delta CT\,(WT)}}$ where ΔC_T mutant is

(C_T of target gene) - (C_T of GAPDH) while ΔC_T WT (C_T of target gene) - (C_T of GAPDH). C_T values for each set of reaction were averaged and standard deviation was calculated. Folds changes of each gene transcript in mutant expressing cell were compared with wild type, considering the level of corresponding transcript of each gene in wild type expressing cells as one.

Validation of HSP70 at protein level by western blot analysis

Significantly increased level of HSP70 at protein and transcript levels was observed in cells expressing L861Q mutant more selectively in repeated experiments as compared to other mutants and wild type expressing cells. Thus, to further validate the expression of HSP70 at different times. Total RNA and protein lysates were recovered from cells expressing L861Q mutant at every 5min intervals for 15min following EGF stimulation. Real time PCR amplification was done using HSP70 specific primers and 25μg total protein sample was subjected to SDS-PAGE followed by immunoblotting using anti HSP 70 antibody (Thermo Scientific, Pierce).
