# Supplementary material for: Proteomic analysis of HEK293 cells expressing non small cell lung carcinoma associated epidermal growth factor receptor variants reveals induction of heat shock response
Source: Exp Hematol Oncol. 2015 Jun 12;4:16. doi: 10.1186/s40164-015-0010-5 (PMC4490733; doi:10.1186/s40164-015-0010-5)
Supplement: Additional file 2: — EGFR mutation analysis on lung tumor tissues. [file 40164_2015_10_MOESM2_ESM.pdf]

Additional file 2: Mutational sequences of exons encoding EGFR tyrosine kinase domain.

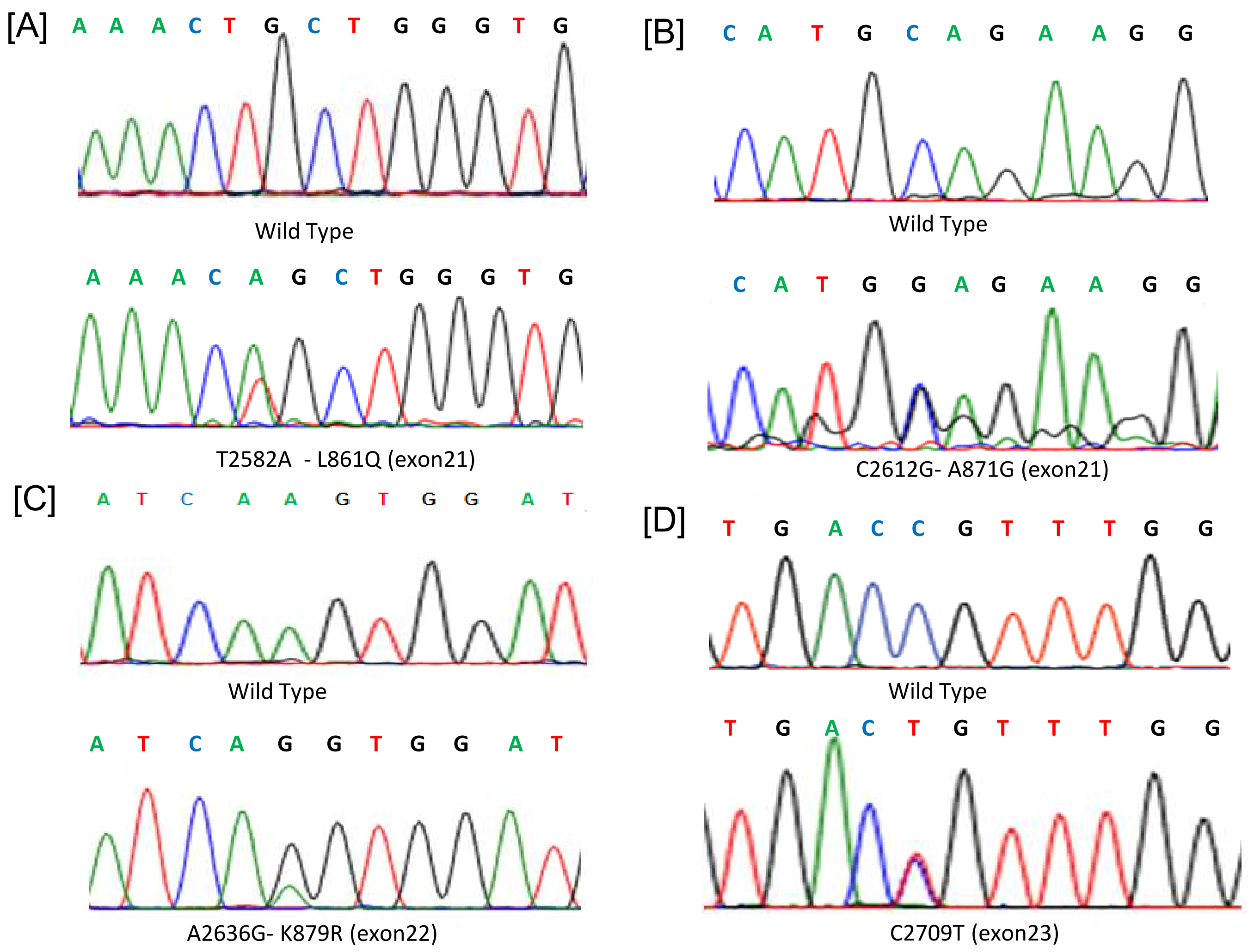

A and B. Base changes indicated as double peaks at positions 2582 (T to A), 2612 (C to G) in exon21. C. Base change at position 2636 (A to G) in exon22 . D. The change at position 2709 (C to T) in exon23 .
