# Supplementary material for: Proteomic analysis of HEK293 cells expressing non small cell lung carcinoma associated epidermal growth factor receptor variants reveals induction of heat shock response
Source: Exp Hematol Oncol. 2015 Jun 12;4:16. doi: 10.1186/s40164-015-0010-5 (PMC4490733; doi:10.1186/s40164-015-0010-5)
Supplement: Additional file 3: — Ligand dependent phosphorylation of EGFR mutants. [file 40164_2015_10_MOESM3_ESM.pdf]

Additional file 3 : **Ligand dependent phosphorylation of mutants' *vs.* wild type receptor**

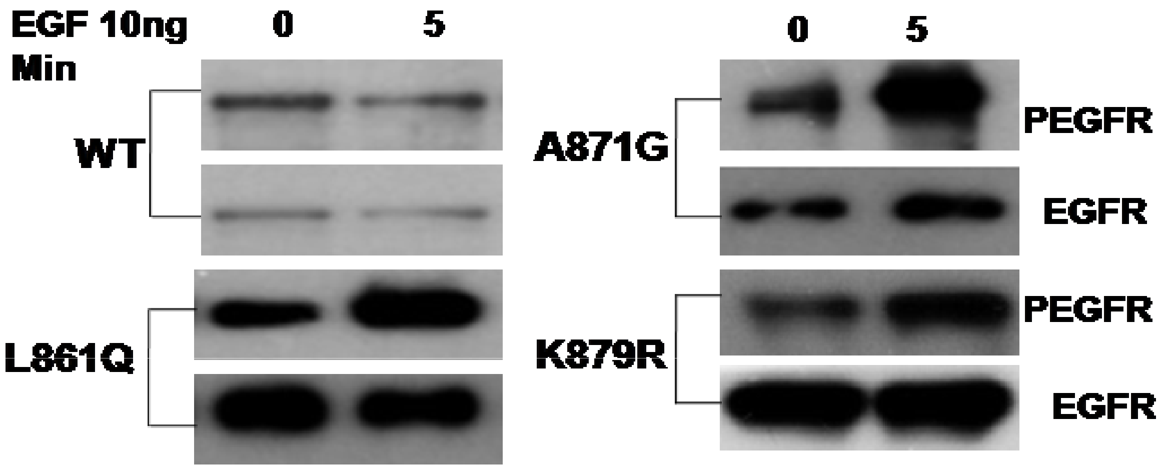

HEK293 cells transfected with wild type and mutant receptors independent of each other were serum starved overnight followed by EGF stimulation. At 10 min post stimulation, total protein from each cell type was recovered and immunoblotted with anti phospho EGFR antibody. Blots were stripped and re-probed with total EGFR antibody
