# Supplementary material for: Proteomic analysis of HEK293 cells expressing non small cell lung carcinoma associated epidermal growth factor receptor variants reveals induction of heat shock response
Source: Exp Hematol Oncol. 2015 Jun 12;4:16. doi: 10.1186/s40164-015-0010-5 (PMC4490733; doi:10.1186/s40164-015-0010-5)
Supplement: Additional file 4: — Sensitivity of mutant receptors to Gefitinib. [file 40164_2015_10_MOESM4_ESM.pdf]

Additional file 4: Effect of Gefitinib on HEK 293 expressing wild type and mutant receptors, L861Q and A871G

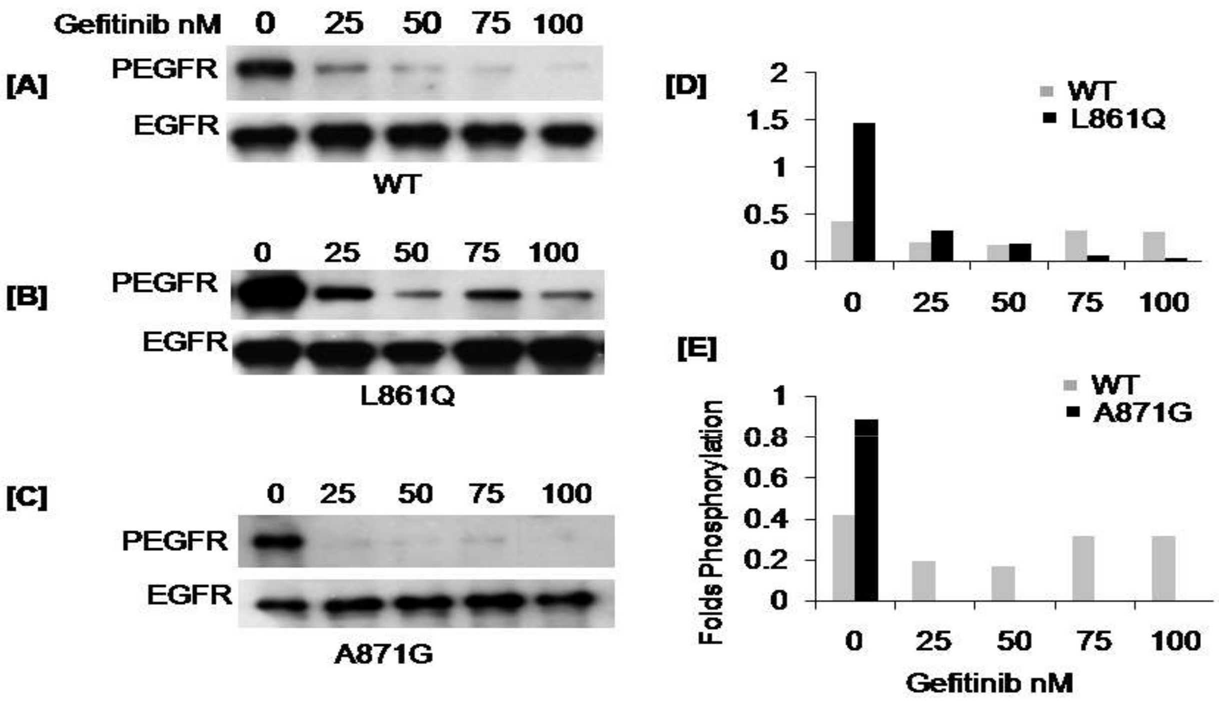

HEK293 cells expressing mutant or wild type receptor were serum starved overnight and treated with different concentrations of Gefitinib drug (TKI) for 60min followed by EGF stimulation for 10min. Lysates were prepared and receptor phosphorylation was measured. A. Phospho and total EGFR levels of wild type receptor. B & C. Phospho and total EGFR levels of each mutant L861Q and A871G respectively. D &E. Densitometry values of signal intensity of each mutant *vs*. wild type receptor.
