# Supplementary material for: Proteomic analysis of HEK293 cells expressing non small cell lung carcinoma associated epidermal growth factor receptor variants reveals induction of heat shock response
Source: Exp Hematol Oncol. 2015 Jun 12;4:16. doi: 10.1186/s40164-015-0010-5 (PMC4490733; doi:10.1186/s40164-015-0010-5)
Supplement: Additional file 5: — Sensitivity of mutant receptors to Erlotinib. [file 40164_2015_10_MOESM5_ESM.pdf]

Additional file 5: Effect of Erlotinib on HEK 293 expressing wild type and mutants, L861Q and A871G

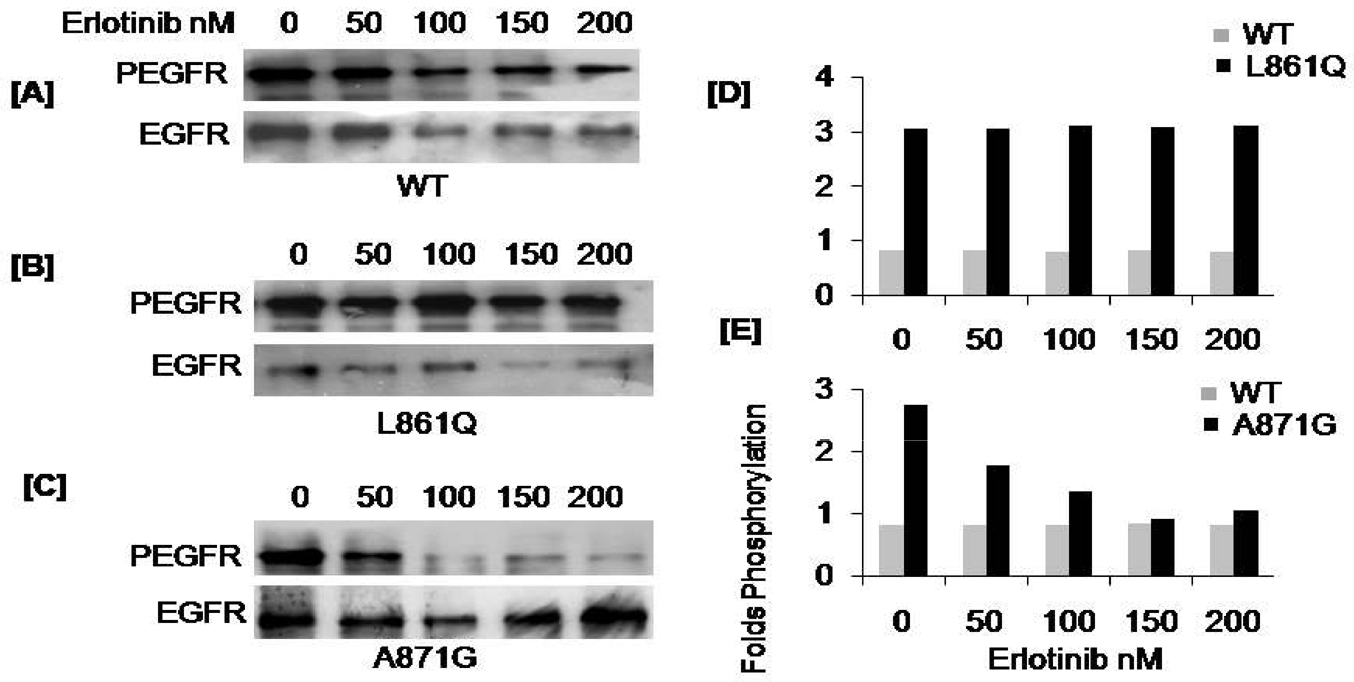

Experiment was done as described in the above figure, but cells were treated with different concentrations of Erlotinib (TKI). A. Phospho and total EGFR levels of wild type receptor. B &C. Phospho and total EGFR levels of each mutant L861Q and A871G respectively. D &E. Densitometry values of signal intensity of each mutant *vs*. wild type receptor
