# Supplementary material for: Proteomic analysis of HEK293 cells expressing non small cell lung carcinoma associated epidermal growth factor receptor variants reveals induction of heat shock response
Source: Exp Hematol Oncol. 2015 Jun 12;4:16. doi: 10.1186/s40164-015-0010-5 (PMC4490733; doi:10.1186/s40164-015-0010-5)
Supplement: Additional file 6: — 2D gel with resolved proteins of EGFR expressing cells. [file 40164_2015_10_MOESM6_ESM.pdf]

Additional file 6: Representative 2D gel with resolved protein spots of wild type EGFR expressing HEK293 cell lysates

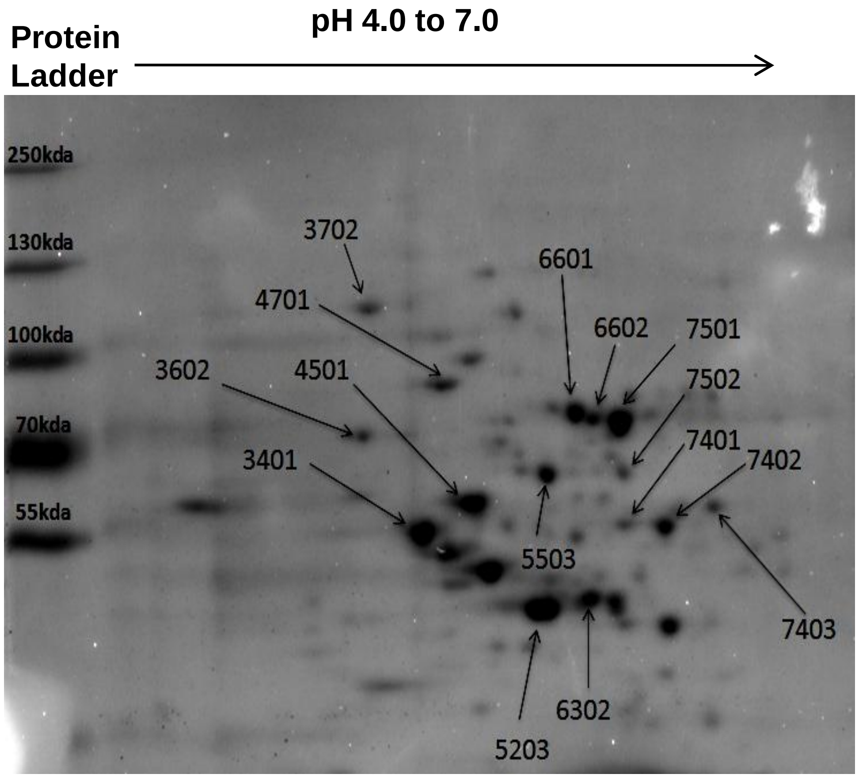

Protein lysates were recovered from cells expressing all three mutants, L858R, L861Q, A871G and wild type receptor independent of each other following overnight serum starvation and 10min EGF stimulation. Lysates were subjected to isoelectric focusing using IPG strips of 4-7pH range followed by 2D gel electrophoresis. Spots analyzed by mass spectrometry are marked and numbered.
