# Supplementary material for: Proteomic analysis of HEK293 cells expressing non small cell lung carcinoma associated epidermal growth factor receptor variants reveals induction of heat shock response
Source: Exp Hematol Oncol. 2015 Jun 12;4:16. doi: 10.1186/s40164-015-0010-5 (PMC4490733; doi:10.1186/s40164-015-0010-5)
Supplement: Additional file 7: — Different densities of protein spots in cells expressing mutants vs. wild type receptor. [file 40164_2015_10_MOESM7_ESM.pdf]

Additional file 7: Different densities of analyzed protein spots of wild *vs*. all three mutants resolved on 2D gels.

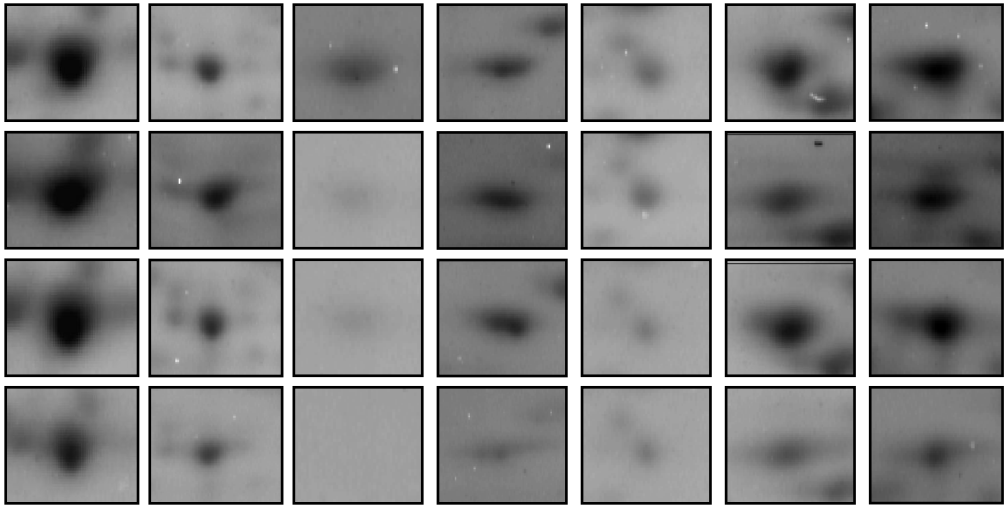

Proteins identified by MS analysis, corresponding protein name and density observed in each mutant vs. wild type receptor expressing cell lysates
