# Supplementary material for: Proteomic analysis of HEK293 cells expressing non small cell lung carcinoma associated epidermal growth factor receptor variants reveals induction of heat shock response
Source: Exp Hematol Oncol. 2015 Jun 12;4:16. doi: 10.1186/s40164-015-0010-5 (PMC4490733; doi:10.1186/s40164-015-0010-5)
Supplement: Additional file 8: — List of proteins differentially regulated in response to mutant L861Q vs. wild type receptor identified by MS analysis. [file 40164_2015_10_MOESM8_ESM.pdf]

Additional file 8: Identification of proteins differentially abundant HEK293 cells expressing L861Q mutant vs. wild type by MALDI-TOF/MS analysis (IEF carried out with 3-10pH range IPG strips)

Spot	Protein name	Accession No	Molecular Weight	PI	Mascot Score	Folds change LQ *vs*. WT
1	Fibrinogen gamma	gi|70906437	50092	5.54	34	1.95
2	Coiled-coil domain-containing protein 54	gi|14211937	37857.69	8.46	25	Undetectable in WT
3	Transcription factor HOXD11	gi|8777659	35500.67	9.43	29	- do-
4	Homeobox protein Hox-B4	gi|13273315	27604.35	9.82	24	-do-
4	SRRM2 protein	gi|116283923	22229.14	9.37	24	-do-
5	DEAD (Asp-Glu-Ala-Asp) box polypeptide 6, isoform CRA_a	gi|119587816	36194.04	8.14	25	-do-
6	Chain A, Crystal Structure Of Human Glycine N-Methyltransferase	gi|55669632	32611.07	6.58	20	- do-
7	IFIT2	gi|116283326	48070.11	8.41	36	2.11
8	dystrobrevin-gamma	gi|1255993	58898.43	8.47	30	2.2
9	Heat shock 70 kDa protein 1A/1B	gi|167466173	70052.23	5.47	31	1.95
10	WD repeat-containing and planar cell polarity effector protein fritz homolog	gi|111548652	85083.71	5.96	28	2.11
