# Supplementary material for: Proteomic analysis of HEK293 cells expressing non small cell lung carcinoma associated epidermal growth factor receptor variants reveals induction of heat shock response
Source: Exp Hematol Oncol. 2015 Jun 12;4:16. doi: 10.1186/s40164-015-0010-5 (PMC4490733; doi:10.1186/s40164-015-0010-5)
Supplement: Additional file 9: — List of real time PCR primers. [file 40164_2015_10_MOESM9_ESM.pdf]

Additional file 9: Gene specific primer sequences used for real time PCR amplification

S. No.	Gene name	Primer sequence
**1**	Homo sapiens heat shock 70kDa protein 1B (HSPA1B)	FP-CCACCAAGCAGACGCAGAT<br>RP-GCCCTCGTACACCTGGATCA
**2**	Homo sapiens fibrinogen gamma chain (FGG)	FP-TGGATCTGGTTGGTGGATGA<br>RP-TTTTGAGTAAGTGCCACCTTGGT
**3**	Homo sapiens interferon- induced protein with tetratricopeptide repeats 2 (IFIT2)	FP-AAAGCTGATGAGGCCAATGATAA<br>RP-GCATGGAGGCTGGCAAGA
**4**	Homo sapiens homeobox D11 (HOXD11)	FP-CTTCGCTAGCAAGCCTTCGT<br>RP- CGGAGCCAGGTTGGAAGAG
**5**	Homosapiens DEAD box helicase 6 (DDX6), transcript variant 1, mRNA	FP-AGCAACAGATGAACCAGCTGAAA<br>RP-AATAGTGGTGGTCATACTCTGTGCTT
**6**	Homo sapiens tubulin, β 2C(TUBB2C), mRNA	FP-GGAGTACCCAGACAGGATCATGA<br>RP-TGTAGGGCTCCACCACTGTGT
**7**	Homo sapiens tubulin, α 1b (TUBA1B), mRNA	FP-TGACCTTGTGTTGGACCGAAT<br>RP-GAAAACCAAGAAGCCCTGAAGAC
**8**	Homo sapiens chaperonin containing TCP1, subunit 5 (epsilon) (CCT5),	FP-GGCTGCTCGTGTTGCTATTG<br>RP-GGGTTCGGTGTCCTTTATGTCA
**9**	Homo sapiens heat shock70kDa protein (HSPA5)	FP-TGCGTCGGCGTGTTCA<br>RP-CGACATAGGACGGCGTGAT
**10**	Homo sapiens heat shock protein 90kDa beta (Grp94), member 1 (HSP90B1)	FP-CCGCCTTCCTTGTAGCAGAT<br>RP-CCTCTTGGGTCAGCAATTACAGA
**11**	Homo sapiens glyceraldehyde-3-phosphate dehydrogenase	FP-TGCACCACCAACTGCTTAGC<br>RP-GGCATGGACTGTGGTCATGAG
