# Supplementary material for: Proteomic analysis of HEK293 cells expressing non small cell lung carcinoma associated epidermal growth factor receptor variants reveals induction of heat shock response
Source: Exp Hematol Oncol. 2015 Jun 12;4:16. doi: 10.1186/s40164-015-0010-5 (PMC4490733; doi:10.1186/s40164-015-0010-5)
Supplement: Additional file 10: — Quantitative real time PCR of regulated gene transcripts in cells expressing EGFR mutants. [file 40164_2015_10_MOESM10_ESM.pdf]

Additional file 10: Folds difference of wild type *vs.* mutant receptor regulated gene transcripts in HEK293cells

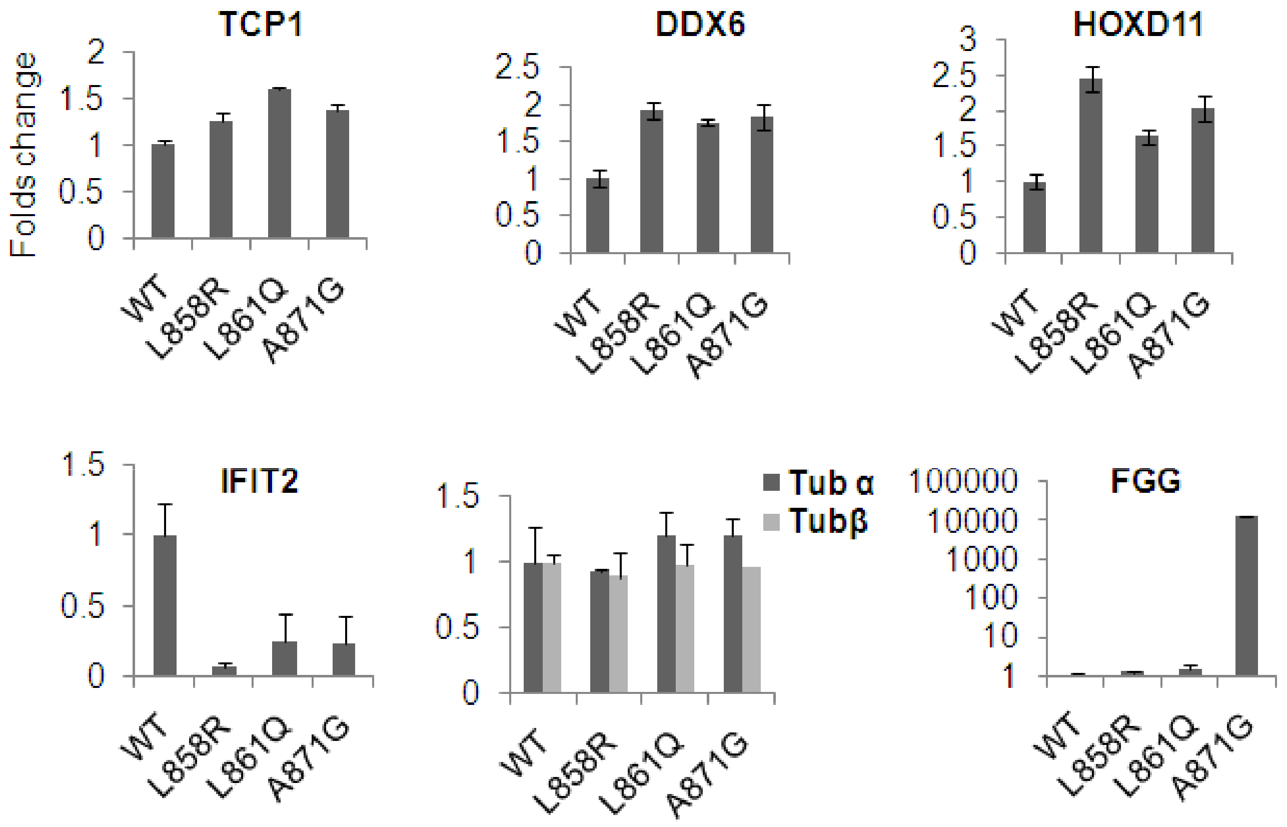

Cells expressing mutants and wild type receptors independent of each other were serum starved overnight followed by EGF stimulation. At 10min post stimulation, total RNA was extracted, reverse transcribed and cDNA was subjected to real time PCR amplification using gene specific primers. Expression level of each gene was normalized to that of GAPDH expression.
